# Intrinsic Dissolution Modeling: Interdependence Between Dissolution Rate, Solubility, and Boundary Layer Thickness

**DOI:** 10.3390/pharmaceutics17050570

**Published:** 2025-04-25

**Authors:** Amelie Marie Mattusch, Gerhard Schaldach, Jens Bartsch, Markus Thommes

**Affiliations:** Laboratory of Solids Process Engineering, Department of Biochemical and Chemical Engineering, TU Dortmund University, Emil-Figge-Str. 68, 44227 Dortmund, Germany

**Keywords:** dissolution rate, solubility, Reynolds number, hydrodynamics, boundary layer, flow channel, IDR, diffusion, surface reaction

## Abstract

**Background/Objectives**: In the past, many drug release models have been presented which attempt to describe the interaction of drugs and excipients in a formulation. Nevertheless, modeling the intrinsic dissolution behavior is essential for understanding the fundamental dissolution mechanisms of drugs and for enhancing the quality of computational approaches in the long term. **Methods**: In this study, the intrinsic dissolution of various pharmaceutical model substances (benzocaine, carbamazepine, griseofulvin, ibuprofen, naproxen, phenytoin, theophylline monohydrate, and trimethoprim) was investigated in dissolution experiments, taking into account the flow conditions in a dissolution channel apparatus. A practicable and generally valid representation was identified to describe the diffusion properties of the drugs in terms of the boundary layer thickness without considering the particle size distribution, physical state, or viscoelastic properties. This representation was supported by numerical simulations using a high-resolution mesh. The influence of the topography on the modeling was also examined. **Results**: Besides the prediction of the influence of a surface reaction limitation or the solubility of a diffusion controlled drug, the boundary layer thickness at the tablet surface is modellable in terms of a freely selectable length and as a function of the diffusion coefficient, drug solubility, and the flow velocity of the dissolution medium. **Conclusions**: Using different methods and a large dataset, this study presents a modeling approach that can contribute to a deeper understanding of intrinsic dissolution behavior.

## 1. Introduction

The release of drugs from pharmaceutical formulations, and thus their efficacy, is influenced by the physicochemical properties of the active ingredients and excipients, as well as the dosage form [1,2,3]. Controlled drug delivery systems have led to the development of numerous models to describe dissolution processes, such as those for polymer matrix systems [2,3,4,5,6]. These models enhance the understanding of drug release patterns, aiding the design of effective formulations [2,6]. Therefore, a comprehensive understanding of intrinsic dissolution mechanisms is needed, considering the pure drug and various influencing factors, such as its solid state and solubility, temperature, hydrodynamics, and the composition and pH of the dissolution medium [2,7,8,9].

In general, the dissolution process describes mass transport through a fluid–solid interface [10] and, therefore, represents a reaction that can be surface-reaction-limited [11,12]. However, it was discovered several decades ago that in most cases, the dissolution of solids in liquids is diffusion controlled [13]. Effectively, during dissolution, a diffusion boundary layer is formed. This is classically described in pharmaceutical technology as the stagnant layer model assuming steady state conditions, based on Noyes and Whitney as well as Nernst and Brunner [13,14,15]. It has been reported in the literature that even for low velocities (*Re* ≥ 10), convection has a greater influence on transport than diffusion, and that diffusion is, therefore, more relevant near the wall surface where the convection flow is virtually non-existent [13,16]. It is, therefore, more appropriate to fundamentally distinguish between different boundary layers during dissolution under laminar flow conditions: a hydrodynamic boundary layer (arising from flow) and a concentration boundary layer (arising from the substance and influenced by flow) [10,11,13]. The concentration boundary layer plays a critical role in dissolution mechanisms, as an increase in its thickness promotes a diffusion-controlled process by reducing the driving force, specifically the concentration gradient [17,18,19]. It can, therefore, also be referred to as a dissolution layer.

Experimentally investigating dissolution under varying conditions can be time-consuming and costly, highlighting the value of predictive theoretical models [3,20]. However, it is emphasized that experimentally standardized conditions, which are necessary for robust mathematical relationships, rarely exist, resulting in a more frequent use of dissolution tests in practice than mathematical models [21]. The flow velocity, geometry of the apparatus, and geometry and positioning of the specimen affect the hydrodynamics in the system [17,22], with the Reynolds number (*Re*) serving as a key descriptor of the flow behavior [8,23,24]. Hydrodynamic limitations of the standard pharmacopeial apparatus have been identified, prompting the proposal of flow channel systems for intrinsic dissolution studies [8,24]. However, even in flow channels, spatial heterogeneity and differences in topography can influence the boundary layer formation and the measurement of dissolution rates [18].

Additionally, alternative methods for determining the intrinsic dissolution rate (*IDR*) have been introduced, such as suspension-based methods and single-particle approaches [25,26,27,28]. A key advantage of these methods is their minimal material requirement. However, certain drawbacks must be considered, including the lack of precise knowledge about relative velocities, and, consequently, the boundary layer thickness around the particles. Furthermore, the increased complexity in modeling due to changes in the particle size, particle size distribution, and morphology is non-negligible [16,29]. In single-particle studies by Andersson et al., a power-law approach has been introduced to model the boundary layer thickness, accounting for the dependence on the particle radius [16]. However, due to the complexity of particle shapes, validation remains challenging. For spherical particles, the same study also investigated the influence of different fluid velocities and proposed a power-law approach, similar to the well-established Sherwood correlations used in mass transfer modeling [16,24,30]. In general, such studies highlight the potential for gaining a fundamental understanding of dissolution processes. However, due to the complexity of experimental setups, modeling approaches, and the range of examined conditions, many dissolution phenomena remain insufficiently understood.

From previous studies, it can be concluded that a hydrodynamically robust dissolution apparatus has been developed and that initial modeling approaches have considered the theory of boundary layer formation [17]. However, these considerations have so far not examined the microscopic level of dissolution in detail, which includes the quantification of the boundary layer thickness and the discussion of surface reactions.

Therefore, the aim of the present study was to derive a novel model for predicting boundary layer thicknesses and solubilities through standardizable intrinsic dissolution tests in a flow channel, using a specimen composed of a compacted pure substance with low porosity, under the assumption of a flat and constant surface, without considering the primary particle size [19,25,31]. Experimental data were correlated with solubility and diffusion coefficients using a model based on Nernst–Brunner, taking into account a recently found sample size’s harmonization [17]. The novel model for determining the dissolution layer thickness enables the extrapolation of boundary layer values for various sample diameters based on a single dissolution test. Furthermore, relationships were established between the dissolution layer thickness, intrinsic dissolution rate, and Reynolds number under steady-state conditions. These are used to discuss dissolution mechanisms, such as the surface reaction at the microscopic level of dissolution, over a broader Reynolds number range under laminar conditions than previously investigated [16,24]. Additionally, computational fluid dynamics (CFD) simulations were conducted to examine boundary layer thicknesses that are experimentally inaccessible, enabling a comparison of real intrinsic dissolution tests with model predictions and validating the proposed model. This novel dissolution modeling approach at the micrometer scale additionally examines the surface topography to evaluate the validity of the flat surface assumption despite mass removal during dissolution, an aspect that has not been thoroughly investigated in previous studies.

## 2. Materials and Methods

### 2.1. Materials

**Figure 1:**
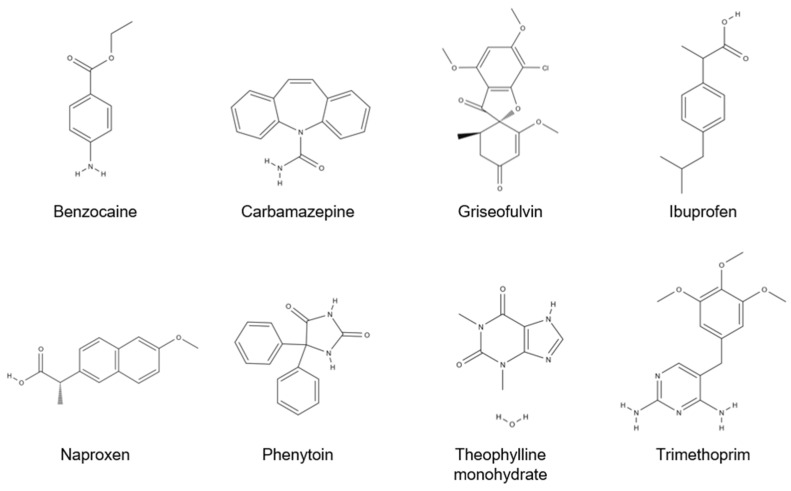
Chemical structures of the drugs used in this work.

The investigated drugs (Figure 1), benzocaine (Caelo, Hilden, Germany), carbamazepine form III (BASF, Ludwigshafen, Germany), griseofulvin (Fagron Services, Uitgeest, The Netherlands), ibuprofen (Fagron Services, Uitgeest, The Netherlands), naproxen (Angene International Limited, Nanjing, China), phenytoin (Recordati, Mailand, Italy), theophylline monohydrate (BASF, Ludwigshafen, Germany), and trimethoprim (Tokyo Chemical Industry, Tokyo, Japan), were used as pure model substances without modification or purification. Filtered and deionized water (Milli-Q Advantage A 10 System; Merck KGaA, Darmstadt, Germany) was utilized as dissolution medium.

The substances are small molecules with molecular weights ranging from 165 to 353 g mol^−1^ and have been utilized in various studies focused on determining intrinsic dissolution rates [3,8,16,17,24,27,31,32]. Except for theophylline monohydrate, these compounds fall into Biopharmaceutics Classification System (BCS) Class II (low solubility), while theophylline is classified as BCS Class I (high solubility) [32,33,34,35]. Consequently, the substances represent a broad spectrum for investigation due to their diverse drug classes and solubility characteristics [34].

### 2.2. Saturation Measurement

For each model drug, a supersaturated solution was prepared and stored for 48 h at the desired temperature (thermal chamber IPP 30; Memmert, Schwabach, Germany). Undissolved particles were separated using a folded filter (597½ Ø270 mm; Schleicher and Schüler, Dassel, Germany) before the saturation concentration was measured in a suitable dilution via spectrophotometry (Specord200Plus; Analytik Jena, Jena, Germany) at 257 nm (benzocaine), 285 nm (carbamazepine), 296 nm (griseofulvin), 222 nm (ibuprofen), 230 nm (naproxen), 220 nm (phenytoin), 272 nm (theophylline), or 203 nm (trimethoprim).

### 2.3. Dissolution Experiment

The flow channel used for the dissolution experiments was hydrodynamically analyzed and recently introduced in the literature [17]. Due to the long inlet section of about 600 mm, velocities up to 0.12 m s^−1^, corresponding to a Reynolds number (*Re*) of ~850 at 37 °C, can be investigated. All model substances were tested using an 8 mm circular sample at 37 °C. For selected substances, different temperatures (e.g., 25 °C and 50 °C) and an additional diameter of 13 mm were also applied.

For the investigation of the dissolution rate at one targeted operating point (specific *Re* and temperature), 250 mg of pure drug substance was compressed in the 8 mm circular die cavity of the flow channel device using a benchtop hydraulic press (Paul-Otto Weber GmbH, Remshalden, Germany) at 15 bar for about 30 s (4 min for carbamazepine [17,36]) to achieve low-porosity compacts [31]. For each 13 mm sample, 450 mg of pure substance was needed.

In the closed-loop system, 900 mL dissolution medium of preheated, demineralized, and degassed water was fed via a gear pump (Reglo Z digital, Z-1830; Ismatec, Wertheim, Germany) into the flow channel. The dissolved drug was quantified using spectrophotometry (Specord 200 Plus; Analytik Jena, Jena, Germany) in samples that momentarily bypassed the turbulently mixed buffer tank before being returned. A 10 mm cuvette was used for benzocaine, theophylline monohydrate, and trimethoprim, while a 50 mm cuvette was used for carbamazepine, griseofulvin, ibuprofen, naproxen, and phenytoin.

As described in previous publications, all tests were performed and evaluated considering the kinetics of the flow channel and concentration measurement [8,17,24]. The measurement time was defined using the accuracy value of 0.01 specified by the spectrometer manufacturer. The mass flux of the dissolved drug, synonymous with the intrinsic dissolution rate (*IDR*) mentioned in the pharmacopoeia, was calculated afterwards [17,24].

### 2.4. Topography Measurement

The sample is a compact of pure drug substance with one surface exposed to the laminar flowing dissolution media [8]. After compaction, the material may expand, causing the surface to become non-planar with the die wall [37]. During the dissolution test, mass is removed, which changes the sample surface topography. For this reason, topographies were determined offline at three points in time: in the dry state, after the equilibration time, and after the measurement [8,17].

The surface topography was visualized using an optical profilometer with a white light interferometer (MicroProf; Fries Research and Technology, Bergisch Gladbach, Germany). The topography was measured at 300 points along a 10 mm evaluation line in the direction of flow across the tablet’s center.

### 2.5. Simulation Setup

A high-resolution mesh is essential for the simulation of boundary layers, but the calculations are often time-consuming. Various strategies were used to minimize the computational effort for the computational fluid dynamics (CFD) simulations conducted using the CFX 2020 R1 software (ANSYS, Inc., Canonsburg, PA, USA).

**Figure 2:**
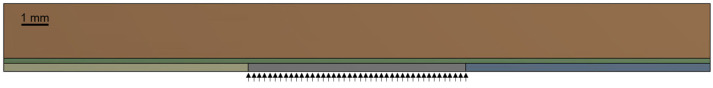
Three-dimensional geometry of the simulated channel with a thickness of 1 µm and a length of 26 mm. The total height of the geometry is 2.5 mm and corresponds to the half channel height from the experiment. The area where the tablet is located is indicated by the arrows. The different colors indicate distinct regions that are connected through interfaces to ensure proper meshing of the geometry.

A thin section half the height of the channel was modeled in 3D with a layer thickness of 1 µm and 26 mm was chosen for the length of the simulated geometry: 9 mm before the tablet, 8 mm for the tablet length, and 9 mm after the tablet. Additionally, only half of the channel height (2.5 mm) was used and the boundary type was set as symmetry (Figure 2). The geometry was modeled with a hexahedron mesh with 3,328,080 elements with an average orthogonal quality of 1.0 ± 1.5·10^−4^ (1 is best) and skewness of 1.1·10^−6^ ± 4.9·10^−4^ (0 is best) [38]. The length of the elements (in the flow direction) was set to 2.5 µm. For the element height, refinement was implemented with a minimum edge length of 0.05 µm at the channel bottom, resulting in a maximum length of 0.38 µm. The aspect ratio for the elements of the geometry varied between 1.3 and 20. The structured mesh with the different regions is connected via interfaces.

The results of the dissolution experiments were used as input variables for the stationary CFD simulation (bulk mass flow rates). The domain type was set as continuous fluid with a homogeneous multiphase model and a laminar flow. The variable composition mixture (water and model substance) was created as liquid with constant properties. The kinematic diffusivity was implemented for the transport equation. At the inlet, a laminar, parabolic flow profile was applied for pure water at 37 °C considering the defined Reynolds number [39]. Dissolution of the drug was modeled by treating the tablet as a drug inlet with a bulk mass flow normal to the boundary at a rate calculated by multiplying the experimental *IDR* with the area of the tablet used in the simulation (square with 1 µm thickness and 8 mm length). A mass fraction of the model substance was set to 1.

A high-resolution scheme with convergence criteria for the mass fraction residual of 10^−6^ was set. The calculations were implemented with double precision and were performed on eighty cores for 48 h.

To evaluate the boundary layer thicknesses, the displacement boundary layer (due to the hydrodynamics of the system) and the concentration boundary layer were considered [40]. The first streamline close to the tablet, which is present in front of the tablet at 0.1 µm from the wall, was used to determine the displacement boundary layer. The concentration boundary layer thickness was defined as 99% of the boundary layer thickness according to the criterion analogous to Blasius’ criterion using the mass fraction of the model substances (Equation (1)) [40,41].

(1) $$w(x,y_{99})=0.99\cdot w(x)$$

## 3. Results and Discussion

### 3.1. Model Approach

An equation for the boundary layer (Equation (2)) was derived based on the intrinsic dissolution rate (*IDR*) according to Ph. Eur. [19,31] and the model according to Nernst–Brunner [14,21], which is valid at a specific running length (*x*). Since forced convection is present, this boundary layer is designated as a dissolution boundary layer (*h*), rather than a diffusion layer. This equation incorporates the dimensionless boundary layer normalization factor (*BNF*_e_), introduced in previous work and based on the two-dimensional boundary layer theory of Blasius, to adequately account for the normalization to the sample area, irrespective of the substance used [17]. Due to its origin, a flat surface is assumed in the model.

(2) $$h(x)=\frac{D\cdot c_{s}}{IDR}\cdot \frac{1}{BNF_{e}}\cdot {\left(\frac{x\;\left[mm\right]}{1000\;\left[mm\right]}\right)}^{0.378}$$

The model fundamentally considers two aspects: the influence of the substance (*D*·*c*_s_/*IDR*) and the influence of the test apparatus (1/*BNF*_e_·(*x*/1000)^0.378^). For a given setup, the solubility (*c*_s_) and the diffusion coefficient (*D*) primarily influence dissolution [41,42].

The diffusion coefficient can be calculated based on the chemical structure at the corresponding temperatures using the modified Stokes–Einstein equation (Equation (3), Table A1).

(3) $$D=\frac{k_{B}\cdot T}{6\pi \cdot \eta \cdot \left(\frac{n_{f}}{6}\cdot R_{vdW}\right)}$$

This takes into account Boltzmann’s constant (*k*_B_), the temperature (*T*), the dynamic viscosity (*η*), a numerical factor of the modified equation (*n*_f_), and the van der Waals radius (*R*_vdW_) [43,44].

Assuming the dissolution boundary layer (*h*) depends solely on the intrinsic dissolution rate (*IDR*), saturation concentration (*c*_s_), and diffusion coefficient (*D*), this model implies that experimental dissolution data (*IDR*) can be utilized in two ways: to fit *c*_s_ values or to determine *h* using a known value of *c*_s_. Since *h* can generally be determined as a function of the Reynolds number (*Re*) according to the Blasius equation [10], it can be assumed that the ratio of substance-specific properties (*c*_s_ and *D*) to the substance-dependent dissolution rate (*IDR*) follows a universal relationship governed by flow conditions and can be characterized by *Re*.

### 3.2. Solubility Fitting

The first approach of the hypothesis, that the model could be applied to fit solubility values from intrinsic dissolution experiments, was pursued. The fundamental assumption underlying this approach is the existence of a master curve capable of describing the dissolution process as a function of *Re*. For this purpose, all dissolution measurement results for one model substance at different temperatures were used to obtain a trendline relating the *Re* to *IDR/c*_s_*/D* for those dissolution tests. Temperature-dependent effects are also taken into account in the *Re* and *D* to account for differences in viscosity or diffusion. The water solubilities (*c*_s_) were determined experimentally at different temperatures and compared with the existing literature values (Table A1).

**Figure 3:**
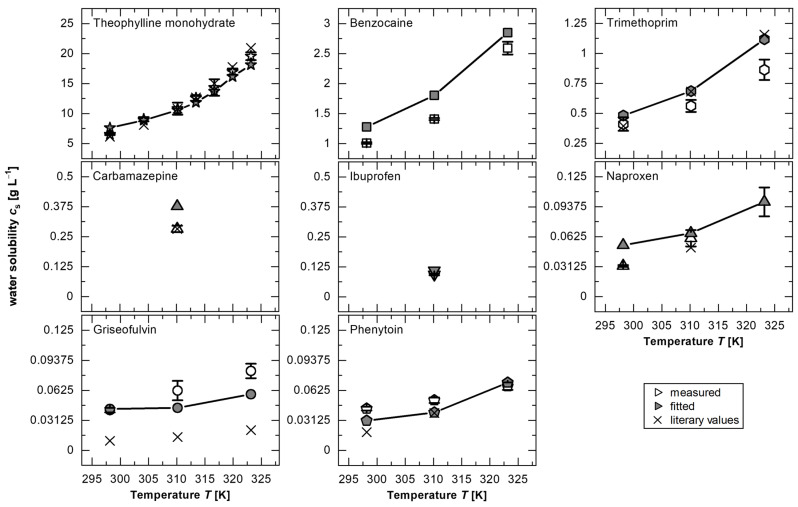
Solubility of model substances (theophylline monohydrate, benzocaine, trimethoprim, carbamazepine, ibuprofen, naproxen, griseofulvin, and phenytoin): measured (white symbols; *av* ± *sd*; *n* = 4), fitted (grey symbols), and literary values (x) for theophylline monohydrate [45], trimethoprim [46], carbamazepine [36], naproxen [47], griseofulvin [9], and phenytoin [48].

For the verification of the hypothesis, theophylline monohydrate was primarily chosen due to the availability in the literature of values at multiple temperatures, providing a sufficiently large dataset to propose and verify a generalizable, substance-independent model. The best agreement was observed for a power function (*IDR/c*_s_*/D* = 2.125·*Re*^0.3^) with a coefficient of determination *R*^2^ = 0.94, indicating a strong agreement between the model and the experimental data. Assuming the validity of this function across all tested temperatures, the fitted solubility (*c*_s,fit_) was determined for each measurement point by minimizing the sum of the squared errors within the dataset corresponding to a specific temperature. The obtained values for *c*_s,fit_ were found to be in excellent agreement with the experimental values of *c*_s_ from this study and the study by Zhang and Rasmuson [45] (Figure 3 and Table A1). The power function fitted to the theophylline monohydrate data was applied to fit *c*_s,fit_ for all other substances, under the assumption that substance-specific properties are fully accounted for by the variables *D* and *c*_s_.

For all substances, this procedure shows that the fitted solubilities (*c*_s,fit_) are in a similar range, and in many cases do not deviate significantly from the measured values, confirming the hypothesis of the existence of a master curve to describe the dissolution process as a function of solubility (Equation (2)).

Furthermore, deviations between *c*_s_ and *c*_s,fit_ may arise from the sample preparation during the experimental determination of *c*_s_. Similar discrepancies can also be present in the literature values, for example, if non-separated particles dissolve during dilution steps or due to differences in dissolution mechanisms, such as diffusion-controlled versus surface-reaction-limited processes [24,49]. To illustrate, a greater influence of the surface reaction was attributed to naproxen from experiments with the rotating disc apparatus [3]. For lower temperatures (25 °C), this could be confirmed due to the significantly different *c*_s,fit_ from the experimentally measured value (Figure 3, Table A1). A dissolution limitation caused by a surface reaction decreases with the increasing temperature due to greater particle mobility and increased diffusion, resulting in no significant deviations in *c*_s_ for, as an example, 50 °C in the investigated *Re* range. Therefore, the deviation in solubility of approximately 20% for theophylline monohydrate at 25 °C could be caused by a dominance of the surface reaction. Additionally, a surface-reaction-limited dissolution mechanism has been discussed in the literature for griseofulvin [24].

### 3.3. Dissolution Modeling

A general verification of the model (Section 3.1) was conducted to assess whether the ratio of *c*_s_ and *D* to *IDR* follows a universal relationship governed by flow conditions and characterized by *Re*, accounting for the test apparatus and different sample sizes. This means that predicting the dissolution boundary layer thickness (*h*), a parameter known to significantly influence the dissolution process [17], is feasible based on intrinsic dissolution tests for cases where *c*_s_ is known.

**Figure 4:**
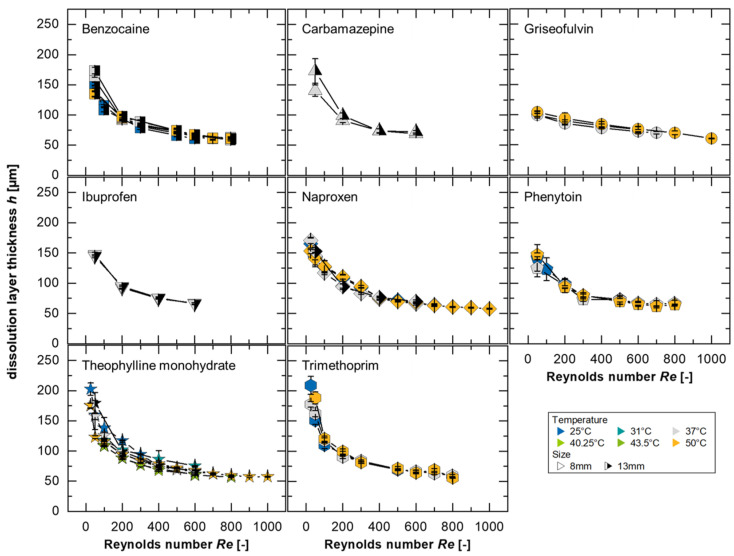
Modeled dissolution data with fitted solubilities of the different substances (symbol-coded) at different temperatures (25–50 °C, color-coded) and using different sample diameters (8 mm: symbol single color, 13 mm: symbol half black; *av* ± *sd*; *n* = 3–8). Each data point represents the average of at least 3 dissolution experiments and was calculated for a tablet length of 8 mm.

In this study, 738 dissolution experiments were performed in a flow channel at different temperatures using eight different substances. The results of the dissolution experiments were modeled using Equation (2) (running length was set to *x* = 8 mm) assuming a constant sample surface using the fitted solubilities (*c*_s,fit_). The calculated dissolution layer thicknesses from these experiments is presented in Figure 4. Based on the developed model, it is expected that all substances will form a master curve if the normalization to the solubility and the diffusion coefficient is sufficient and the convection in the system has no substance-specific influence on the dissolution mechanism.

As described by Nernst and Brunner, the diffusion-based dissolution rate depends on the geometric conditions and the intensity of the convection flow that enhances the diffusion process [15,40]. Accordingly, the modeling indicates thinner boundary layers for higher *Re*, which can be explicitly detected in Figure 4 for all substances supporting the existence of a master curve, as represented by a power function in the form *h* = 471.8·*Re*^−0.31^ with an *R*^2^ = 0.94. For small Reynolds numbers (*Re* < 100), a larger inconsistency can be seen, which could be explained by the flow conditions, particularly by the drag coefficient, which depends on the *Re* [24,50]. Discrepancies may indicate differences in dissolution mechanisms or transport rates if diffusion is not the limiting dissolution mechanism [3,15,24,28].

A differentiation between dissolution mechanisms can be made by comparing prediction data with the power functions. It is generally true that the smaller the values for the pre-factor and the closer the exponent is to 0 (i.e., smaller absolute values of the exponent), the more downwardly offset and flatter the curve will be. This indicates a dissolution mechanism limited by the surface reaction, as it shows a reduced dependence on convection forces. In contrast, a power function of the form *h* = *a*·*Re*^−*b*^, with *b* = 0.5 corresponding to Blasius’ two-dimensional hydrodynamic boundary layer thickness [51], could represent a scenario where no limitation due to the surface reaction is present.

The greatest deviations from the model, for the different substances evaluated, were determined to be for griseofulvin at 25 °C and trimethoprim at 50 °C. Neither of the mentioned cases represents the solubility, the diffusion coefficient, or the *IDR* limits of the investigated substances, as both less and more soluble drugs with lower and higher diffusion coefficients as well as lower and higher *IDR* were examined (Table A1). Therefore, the model’s validity is assumed across the evaluated ranges.

Since the largest deviations were also observed in the solubility fitting for griseofulvin, it is valuable to examine both the measured and fitted values (Figure 3, Table A1): the smallest values for the pre-factor *a* (150.4 and 154.8, respectively) and the smallest absolute values for the exponent *b* (0.108 for both) were determined for 25 °C. This lower dependence of the boundary layer thickness on the Reynolds number or flow velocity, which is more pronounced even at a low *Re*, could be due to the stronger influence of the surface reaction on the dissolution compared to diffusion. This mechanism has already been proposed in the literature for griseofulvin [24].

Larger values of the exponent, which are linked to a greater dependence of the boundary layer on *Re*, can be explained by stronger diffusion effects. For trimethoprim, a 71% higher value for the pre-factor, *a* = 806.7, and 0.39 for the exponent were determined. Interestingly, this value of *b* = 0.39 matches the theoretical exponent derived from the Blasius boundary layer in a three-dimensional configuration [17] and was also discussed in a study with a single-particle dissolution test to consider the influence of the fluid velocity on the layer thickness [16]. This suggests that the influence of the surface reaction should be minimal for this substance compared to the other evaluated compounds, or possibly that there is no surface reaction effect on the intrinsic dissolution of trimethoprim in the investigated *Re* range.

In this study, the investigated compounds could not be explicitly assigned to any class, which underlines the complexity of the dissolution process [31]. However, trends, which can be derived from deviations or differences between the substances, can be used to assume the type of intrinsic dissolution behavior to which the substance belongs. In addition, it can be emphasized that at low flow velocities, the differences in the dissolution mechanism of each substance are larger, and at a higher *Re,* differences seem to become negligible (Figure 4).

This model approach is based on the assumption that the surface is constant and flat. The effects of surface changes due to mass erosion, which must occur as a consequence of mass conservation, have not yet been considered. However, since the model has yielded values for *h* in the range of ~100 µm, it would be reasonable to examine the topography of the sample at a microscopic level.

### 3.4. Sample Surface Topography

In the investigations of the *IDR* and consequently in the model approach, a flat and constant surface is assumed. However, in the real case of substance dissolution, changes of the surface are expected, which are relevant at the microscopic level to dissolution. This erosion of the tablet, caused by hydrodynamically induced shear, could affect local fluid dynamics and, consequently, the intrinsic dissolution rate [8,52]. The sample topography was measured at-line to assess the impact of material removal on modeling.

**Figure 5:**
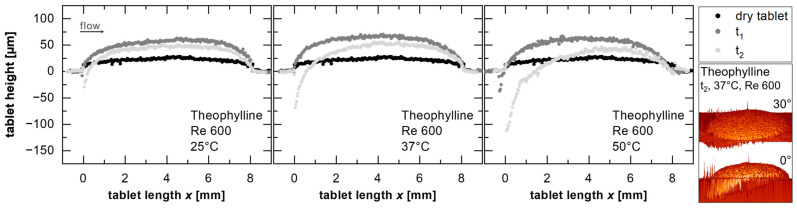
Tablet topography represented by the height of the tablet cross-section perpendicular to the surface, along the diameter of a tablet of theophylline monohydrate at 25, 37, and 50 °C given at three different states: dry tablet (black), start of evaluation period (t_1_ = 1 min, mid-gray), and after dissolution test (t_2_ = total 3 min, light gray) for *Re* = 600. And 3D-view of the tablet (t_2_, 37 °C, *Re* = 600) from a 30° (top) and 0° (bottom) angle.

For the model substance with the highest water solubility, theophylline monohydrate, the topography is represented in Figure 5 by the height of the tablet cross-section perpendicular to the surface, along the diameter of the tablet in the direction of flow for a *Re* = 600 at 25, 37, and 50 °C. The three curves per temperature correspond to the tablet in the dry state prior to the dissolution test, the tablet after the process-related waiting time (t_1_, corresponds to the start of the evaluation period, 1 min [17]), and the tablet at the end of the dissolution test (t_2_, total of 3 min for theophylline monohydrate).

The protruding surface of the tablet in the dry state (Figure 5, black) resulted from elastic recovery after the compression of the material at high forces (~5 kN) and long dwell times (~30 s) to achieve low-porosity compacts [37]. The further protruding of the surface after contact with water (Figure 5, mid-gray) is due to swelling [53]. In this study, the evaluation was conducted considering the flow channel kinetics and concentration measurements under steady-state conditions. In this state, an equilibrium is established between the migration of water into the solid and the migration of the solid into the water, as described in the literature, to characterize the solid–liquid interface velocity during dissolution (velocity of the boundary) [17]. During this phase, the swelling process, where higher transport rates into the tablet occur compared to those leaving it [53], is not considered. Therefore, the topographical differences between t_1_ and t_2_ are attributed to mass removal (Figure 5).

The depression at the tablet’s edge (Figure 5, *x* = 0 mm) is caused by fluid shear forces. At the tablet’s end (*x* = 8 mm), the topographical differences decrease over time (Figure 5, light gray), which may be linked to the hydrodynamics near the tablet (a slanting surface) or a flow-induced growth of the concentration boundary layer, resulting in reduced mass flow rates [17]. For theophylline monohydrate, the greatest change in the surface was due to the removal of mass at the edge of the tablet exposed to the flow (−110 µm at 50 °C, Figure 5). During the dissolution test, the extent of erosion at the tablet’s edge depends on the solubility of the substance, e.g., −50 µm for benzocaine and +33 µm for naproxen (at 50 °C for a *Re* = 600). For naproxen, the positive values were attributed to the swelling. Since the fit of *c*_s_ (Section 3.2) and the representation of a master curve for determining *h* (Section 3.3) were successfully demonstrated for theophylline monohydrate, the influence of tablet erosion can be considered negligible. It is plausible that the influence of topography is adequately accounted for through the normalization to *c*_s_, as otherwise, one would expect larger discrepancies between *c*_s,fit_ and *c*_s_ or a lack of curve overlap when modeling *h* with an R^2^ value of 0.94.

Nevertheless, the tablet heights observed fall within a range where the boundary layers also reside according to the model calculations. The extent to which the topography may influence the modeling, and the validity of the model is examined in the following discussion.

### 3.5. Boundary Layer Simulation

In the model, a dissolution boundary layer was considered and was calculated (Figure 4) to be a similar order of magnitude as the values from the topography measurement (Figure 5). Since boundary layers are challenging to observe directly, simulations provide an effective method of investigating them. Therefore, the boundary layer simulation serves as a tool to validate the model against the corresponding results.

A flat surface was chosen, similar to the model of the unstirred hydrodynamic boundary layer on the solid surface, as described by Blasius [40,41].

**Figure 6:**
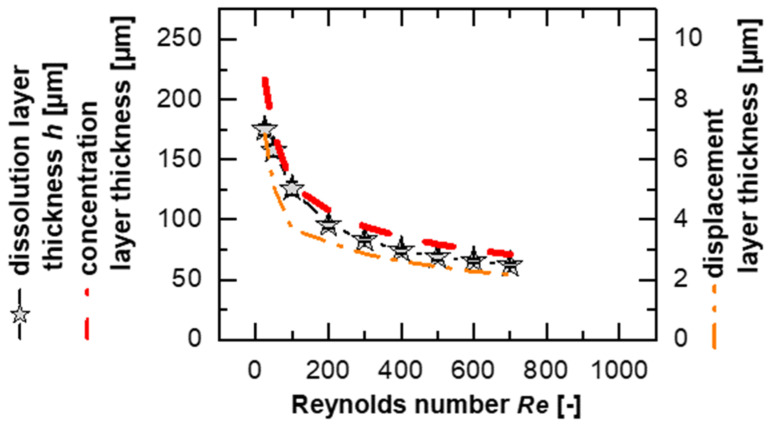
Simulated concentration boundary layer thickness (red dashed line) and displacement boundary layer thickness (orange dash–dotted line) for a flat tablet surface at the tablet’s edge (8 mm) compared to the experimental results of the dissolution layer thickness (symbols) for theophylline monohydrate for different *Re* at 37 °C.

Theophylline was selected as the model substance for validation because it has the highest solubility and highest *IDR* (Table A1), leading to the most pronounced topographical differences. The comparison between the simulation results (Equation (1), concentration layer thickness; Figure 6, red dashed line) and the experimental results (Equation (2), dissolution layer thickness; symbols) only shows an average deviation of 13%, highlighting the applicability of the simulation models. The highest deviation, 24%, is again with the smallest *Re*.

The simulated displacement layer thickness (Figure 6, orange dash–dotted line) correlates to the simulated concentration layer thickness and the modeled dissolution boundary layer, confirming the relationship between the concentration layer and flow in the simulation and experiment. This suggests that material removal at the tablet’s leading edge does not significantly influence the overall behavior, particularly at high *Re*, as larger differences would otherwise be expected (e.g., an additive increase in the boundary layer thickness by approximately 60 µm, corresponding to the maximum protrusion of the tablet at a *Re* = 600 (Figure 5)).

**Figure 7:**
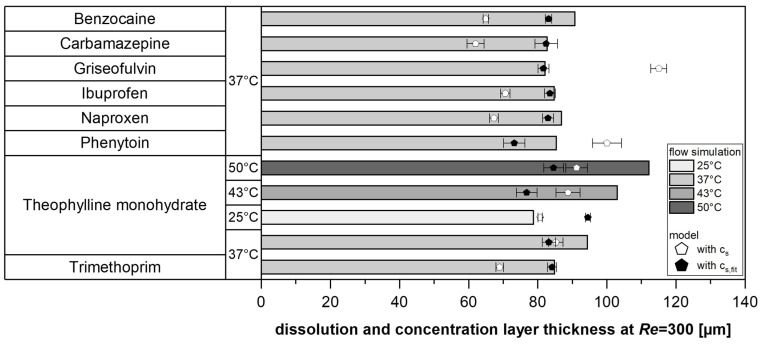
Simulated concentration layer thickness and experimentally modeled dissolution layer thickness with measured (white symbol) and fitted (black symbol) solubilities for tested model substances at the tablet’s edge (8 mm) for *Re* = 300.

To validate the simulation, the concentration layer thickness for various model substances was simulated at a *Re* = 300 and compared with the model values (Equation (2)). Specifically, the measured solubility in water (*c*_s_) was compared with the fitted values (*c*_s,fit_) to assess the robustness of the solubility fitting while simultaneously providing a fundamental verification of the model (Figure 7).

In most cases, the modeled values from the experiments were lower than the simulation results. However, the deviations between the experimentally determined dissolution layer thickness (using fitted solubilities) and the simulated concentration layer thickness were below 10% for approximately half of the tests. Therefore, it could be shown for various substances and temperatures that this model approach, which is based on a master curve, provides comparable values to the use of a simple CFD approach, which uses the experimentally measured *IDR* and the diffusivity *D* of the pure substance as input variables and does not take solubilities into account.

The model serves as a proof of concept for investigating and describing substance-specific differences. These findings also demonstrate the effectiveness of solubility fitting, supporting the use of the proposed model to determine solubility from dissolution experiments (Equation (2), Section 3.2).

### 3.6. Applicability and Remaining Challenges

A good agreement between CFD-simulated and experimentally determined boundary layer thicknesses was observed for the different drugs investigated, indicating that the proposed model can be flexibly applied to compounds of different solubilities.

In this work, a *Re* range from 25 to 1000 was investigated, corresponding to mean flow velocities from 27 to 1071 mm s^−1^ and volume flow rates between 2 and 80 mL min^−1^ at 25 °C. At lower *Re*, larger deviations between different tested drugs were observed, which were attributed to potential dissolution limitations due to surface reaction effects. Therefore, this range may be of particular interest for future research, as surface reaction mechanisms are expected to play a more prominent role and may lead to more pronounced differences between substances. This is particularly relevant when aiming to better understand gastrointestinal fluid flow processes in humans, which have been reported to be around 1 mL min^−1^ [16,54].

A key advantage of the proposed model lies in its ability to estimate the dissolution layer thickness for any sample diameter using the *BNF*_e_, which is based on a power-law relationship [17]. Similarly, in a single-particle dissolution model, power-law correction for the particle radius was introduced [16]. However, this model also required an additional proportionality factor, which the present model avoids, further simplifying its application. A direct comparison with the boundary layer thicknesses obtained in the single-particle dissolution test is challenging due to the discussed discrepancies at lower *Re*. Nonetheless, a comparison can still help explore the applicability range of the proposed model. For instance, in the single-particle dissolution study, a layer thickness of approximately 5 µm was reported for ibuprofen at a flow velocity of 103 mm s^−1^ under room-temperature conditions. Based on the data presented in this study (Table A1), Equation (2) yields a dissolution layer thickness of 7.0 µm for ibuprofen at 37 °C and a *Re* = 100 using *c*_s,fit_, or 6.4 µm when using *c*_s_. We estimate the accuracy and variability of these results to be comparable to those of Andersson et al.’s single-particle approach [16].

Overall, the presented model for determining the dissolution layer thickness demonstrates robustness and precision in its application to modeling under laminar flow conditions in a flow channel setup, especially for a *Re* > 100.

## 4. Conclusions

Conducting dissolution tests in a flow channel, this study provided insights into the microscopic description of the process. The dissolution layer thickness (*h*) can be modeled as a simple power function based on the diffusion coefficient (*D*) and solubility (*c*_s_) and can be mapped using flow simulations independent of solubility. The steady-state approach underlying both methods enables the identification of qualitative differences between substances with minimal experimental and computational effort.

The proposed model can be used to determine the solubilities utilizing simple dissolution experiments, especially with high Reynolds numbers (*Re*), because the approach is based on purely diffusion-controlled dissolution, as described by Nernst–Brunner. Due to the model’s origin, the influence of the surface reaction can be investigated. The comparison of the slope and exponent of the power function used to model the dissolution layer thickness with the Reynolds number provides a consistent basis for interpretation. Nevertheless, no significant differences were found at a low *Re* in this study, which means that diffusion-controlled drugs and surface-reaction-limited drugs cannot be divided into two classes, while emphasizing the complexity of the dissolution mechanisms and the requirements for robust analytical methods.

The results from the topography study also suggest that particle sizes and shapes, as well as swelling and erosion, have a negligible effect on intrinsic dissolution testing. The findings of this study could simplify existing models, facilitate user-friendly dissolution testing in flow channels, support fundamental modeling with validity independent of the tablet or particle size and substance, and improve the quality of computational approaches and formulation development.

## Data Availability

The data that support the findings of this study are available from the corresponding author upon reasonable request.

## Abbreviations and Symbols

The following abbreviations and symbols are used in this manuscript.

**Symbol**	**Description**	**SI Unit**
*a*	Pre-factor of power function	-
*av*	Average	-
*b*	Exponent of power function	-
*BNF*_e_	Boundary normalization factor with exponent e	-
CFD	Computational fluid dynamics	-
*c*_s_	Water solubility	g L^−1^
*c*_s,fit_	Fitted water solubility	g L^−1^
*D*	Diffusion coefficient	m^2^ s^−1^
*h*	Dissolution boundary layer thickness	m
*IDR*	Intrinsic dissolution rate	kg m^−2^ s^−1^
*k*_B_	Boltzmann’s constant	kg m^2^ s^−1^ K^−1^
*n*	Number of experiments	-
*n*_f_	Numerical factor of modified Stokes–Einstein equation	-
R^2^	Coefficient of determination	-
*Re*	Reynolds number	-
*R*_vdW_	van der Waals radius	m
*sd*	Standard deviation	-
*t*	Time	s
*T*	Temperature	K
*w*	Mass fraction	-
*x*	Running length	m
*y*	Ordinate length	m
*η*	Dynamic viscosity	kg m^−1^ s^−1^

## Appendix A

**Table A1:** Material properties and *IDR* data (at *Re* = 100 for a tablet diameter of *x* = 8 mm) for the dissolution tests and details of the saturation concentrations (*c*_s_). Calculation of the diffusion coefficient (*D*) via modified Stokes–Einstein equation (Equation (3)) with van der Waals radius (*R*_vdW_) and estimated numerical factor (*n*_f_) [43] at various temperatures (*T*).

Drug Substance	*R*_vdW_ [Å]	*n*_f_ [-]	*T* [°C]	*D* × 10^−10^ [m^2^ s^−1^]	Measured *c*_s_ [g L^−1^]	Fitted *c*_s_ (*c*_s,fit_) [g L^−1^]	(Interpolated) Literature Solubility Data [g L^−1^]	*IDR* [mg cm^−2^ min^−1^] at *Re* = 100 (107 mm s^−1^, 8 mL min^−1^) for 8 mm Specimen
Benzocaine	3.361	5.4	25	8.18	1.01 ± 0.01	1.18		0.054 ± 0.001
			37	10.94	1.41 ± 0.02	1.69		0.092 ± 0.001
			50	14.44	2.59 ± 0.11	2.66		0.205 ± 0.012
Carbamazepine	3.871	5.7	37	8.99	0.282 ± 0.013	0.348	0.282 [36]	0.017 ± 0.001
Griseofulvin	4.217	5.7	25	6.12	0.042 ± 0.002	0.040	0.010 [9]	0.0016 ± 0.0001
			37	8.18	0.062 ± 0.001	0.041	0.014 [9]	0.0023 ± 0.0001
			50	10.80	0.083 ± 0.008	0.055	0.021 [9]	0.0038 ± 0.0002
Ibuprofen	3.879	5.7	37	8.97	0.093 ± 0.003	0.102		0.0048 ± 0.0001
Naproxen	3.701	5.5	25	7.23	0.032 ± 0.001	0.049		0.0018 ± 0.0002
			37	9.66	0.061 ± 0.009	0.061	0.051 [47]	0.0033 ± 0.0001
			50	12.75	0.099 ± 0.015	0.092		0.0059 ± 0.0005
Phenytoin	3.814	5.6	25	6.89	0.044 ± 0.002	0.031	0.019 [48]	0.0011 ± 0.0002
			37	9.21	0.052 ± 0.003	0.039	0.039 [48]	0.0019 ± 0.0002
			50	12.15	0.067 ± 0.004	0.071		0.0042 ± 0.0001
Theophylline monohydrate	3.194	5.1	25	9.03	6.52 ± 0.12 [8]	7.64	6.14 [45]	0.303 ± 0.041
			31	10.49	9.07 ± 0.36	8.89	8.11 [45]	0.476 ± 0.008
			37	12.07	10.82 ± 1.00 [8]	10.57	10.81 [45]	0.609 ± 0.026
			40.25	13.00	12.59 ± 0.50	11.86	12.68 [45]	0.851 ± 0.011
			43.5	13.93	14.35 ± 1.39	13.70	14.99 [45]	1.045 ± 0.004
			46.75	14.90	17.00 ± 0.52	16.17	17.77 [45]	1.251 ± 0.029
			50	15.93	19.56 ± 0.64	18.14	20.95 [45]	1.492 ± 0.009
Trimethoprim	3.971	5.7	25	6.56	0.411 ± 0.056	0.482	0.39 [46]	0.017 ± 0.001
			37	8.77	0.562 ± 0.045	0.685	0.68 [46]	0.031 ± 0.001
			50	11.57	0.863 ± 0.085	1.117	1.16 [46]	0.064 ± 0.002
